# Membrane related dynamics and the formation of actin in cells growing on micro-topographies: a spatial computational model

**DOI:** 10.1186/s12918-014-0106-2

**Published:** 2014-09-09

**Authors:** Arne T Bittig, Claudia Matschegewski, J Barbara Nebe, Susanne Stählke, Adelinde M Uhrmacher

**Affiliations:** 1 Modeling and Simulation Group, Institute of Computer Science, University of Rostock, Albert-Einstein-Str. 22, 18059, Rostock, Germany; 2 Department of Cell Biology, University Medical Center Rostock, Schillingallee 69, 18057, Rostock, Germany; 3 Present address: Agronomy and Crop Science, Faculty of Agricultural and Environmental Sciences, University of Rostock, Rostock, Germany

**Keywords:** Actin filaments, Micro-structured surface, Spatial simulation, Rule-based modeling

## Abstract

**Background:**

Intra-cellular processes of cells at the interface to an implant surface are influenced significantly by their extra-cellular surrounding. Specifically, when growing osteoblasts on titanium surfaces with regular micro-ranged geometry, filaments are shorter, less aligned and they concentrate at the top of the geometric structures. Changes to the cytoskeleton network, i. e., its localization, alignment, orientation, and lengths of the filaments, as well as the overall concentration and distribution of key-actors are induced. For example, integrin is distributed homogeneously, whereas integrin in activated state and vinculin, both components of focal adhesions, have been found clustered on the micro-ranged geometries. Also, the concentration of Rho, an intracellular signaling protein related to focal adhesion regulation, was significantly lower.

**Results:**

To explore whether regulations associated with the focal adhesion complex can be responsible for the changed actin filament patterns, a spatial computational model has been developed using ML-Space, a rule-based model description language, and its associated Brownian-motion-based simulator. The focus has been on the deactivation of cofilin in the vicinity of the focal adhesion complex. The results underline the importance of sensing mechanisms to support a clustering of actin filament nucleations on the micro-ranged geometries, and of intracellular diffusion processes, which lead to spatially heterogeneous distributions of active (dephosphorylated) cofilin, which in turn influences the organization of the actin network. We find, for example, that the spatial heterogeneity of key molecular actors can explain the difference in filament lengths in cells on different micro-geometries partly, but to explain the full extent, further model assumptions need to be added and experimentally validated. In particular, our findings and hypothesis referring to the role, distribution, and amount of active cofilin have still to be verified in wet-lab experiments.

**Conclusion:**

Letting cells grow on surface structures is a possibility to shed new light on the intricate mechanisms that relate membrane and actin related dynamics in the cell. Our results demonstrate the need for declarative expressive spatial modeling approaches that allow probing different hypotheses, and the central role of the focal adhesion complex not only for nucleating actin filaments, but also for regulating possible severing agents locally.

## Background

### Biological motivation

Cells at the interface to an implant surface are able to sense mechanical and biochemical changes in their environment, for instance induced by the interaction with chemical and topographical characteristics of the biomaterial surface via their focal contacts [1]. According to the distinct physico-chemical properties of the biomaterial surfaces, cells have the capacity to adapt to it via cell-specific morphological [2]-[4] and functional aspects, e.g., changes in cell morphology, intracellular architecture of adhesion components [5]-[7] and/or gene and protein expression pathways. For bone cells that were growing on titanium surfaces with regular micro-geometry (namely pillars or grooves), an adaptation of extracellular and intracellular phenotypic traits, including significantly emerging actin filament patterns, has been shown [8],[9].

**Figure 1:**
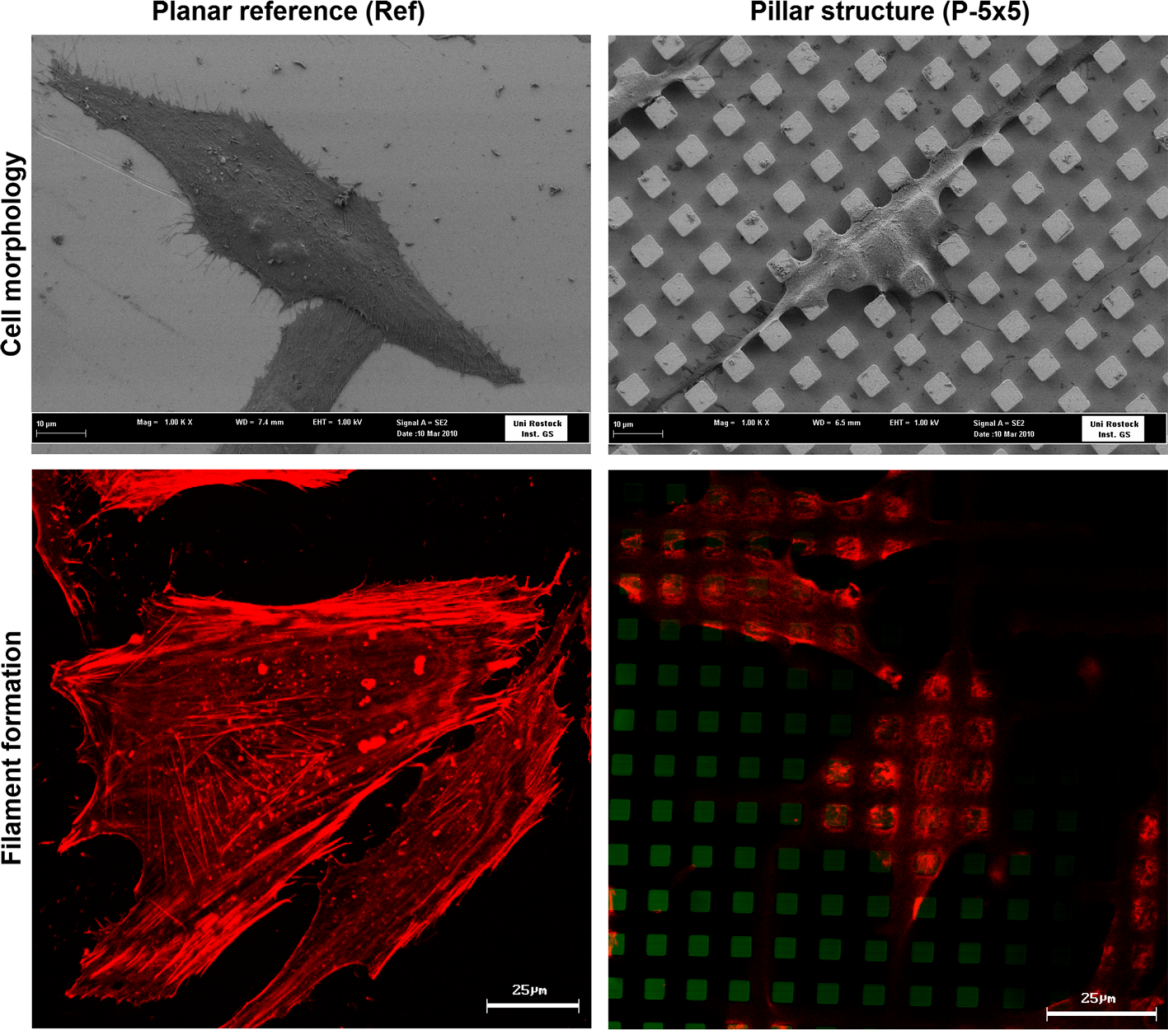
**Cell morphology (top) and formation of actin filaments (bottom) of MG-63 osteoblasts on planar (left) and geometrically micro-pillared (right) titanium surfaces after 24 h.** On the planar surface cells are closely attached to the surface with their entire cell body and exhibit a flattened phenotype. On the pillared surface P-5 × 5 × 5, cells are elongated and their adhesion is mainly restricted to the surface plateaus (FE-SEM Supra 25, Carl Zeiss; bar = 10 *μ*m), image in cooperation with Regina Lange, Institute of Electronic Appliances and Circuits, University of Rostock). Long and well defined filaments actin filaments form in cells on planar surfaces, whereas on the pillared structure P-5 × 5 × 5 the actin filaments are accumulated on top on the pillars in short fibers (LSM 410, Carl Zeiss, green: reflexion mode from the surface, red: phalloidin-TRITC for actin) [9].

It could be recognized in diverse experiments that expression and appearance of intracellular structures as well as overall cell shape are influenced by diverse environmental parameters, especially the physical and geometrical properties of the extracellular matrix, e.g., rigidity, dimensionality, composition and ligand spacing [10]-[12]. In our experiments human osteoblasts rearrange their actin cytoskeleton in typical patterns mimicking the underlying micro-topography (5 *μ*m dimensions, material surface in Figure 1 right). Those changes have so far emerged independently on several chemical cues and variations [13], e.g., usage of glass instead of the bulk material titanium, micro-structured titanium surfaces modified with (i) fibronectin layer due to fetal calf serum, (ii) collagen I coating of the pillars, (iii) sputtering with gold [14] as well as (iv) deposition of a plasma polymer nanolayer exhibiting positively charges to cells [7],[15]. However, the mechanisms behind this restructuring of the actin-cytoskeleton are not clear.

**Figure 2:**
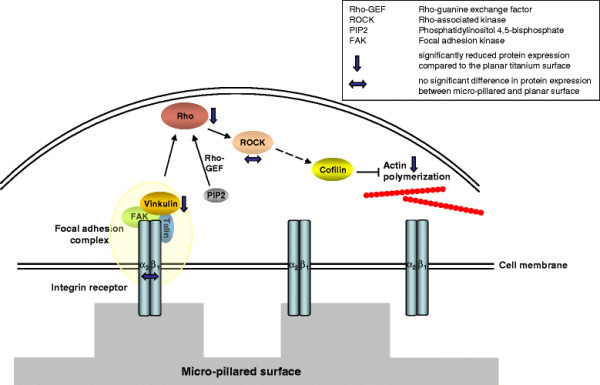
**Simplified schema of signal transduction by which the topographical influence of the surface activates cofilin via the integrin-Rho-ROCK-pathway, finally inhibiting the actin polymerization.** The blue arrows express the total protein expression on the micro-pillared surface in relation to the planar reference based on the biological data obtained from the wet-lab experiments (see Results).

Actin is an abundant and highly conserved eukaryotic cellular protein and the major cytoskeletal component in all eukaryotic cells. It plays a central role in important cellular processes, comprising the transduction of extracellular forces and tensions to the nucleus as well as cell spreading and migration processes [16]. In particular, many studies report that the structural arrangement of the actin cytoskeleton is decisive for subsequent cellular events [17], like the length control of cells or protein expression pathways [18]. Actin exists in two forms: as globular/monomeric G-actin and filamentous/polymeric F-actin, which is self-assembled in linear filaments containing +/- ends (also known as barbed and pointed ends, respectively) as growing and shrinking sites. The dynamic equilibrium of the continuous reorganization of the actin network is based on its controlled polymerization and depolymerization events, which are guided by a complex interplay of actin with a huge number of regulatory molecules (131 listed in [19]). Among those, ADF/cofilin, Rho and ROCK [20] play a central role. These sustain the balance of actin polymerization and depolymerization and therefore are strongly controlled in their expression and activity pattern [16],[18],[21]. Here, the actin regulatory protein, actin depolymerization factor (ADF)/cofilin acts as a key player by severing filaments [22]. ADF/cofilin becomes inactive when it is phosphorylated at its serine 3 residue. ADF/cofilin activity is controlled by the Rho family of small GTPases. Thereby the recruitment of Rho small GTPase and Rho-associated protein kinase (ROCK) leads to subsequent ADF/cofilin phosphorylation [23] (see also Figure 2). Also, PIP2 has been shown to influence the activity of ADF/cofilin via different mechanisms, including a competitive binding of actin and PIP2 on cofilin [21],[24].

Integrins, as transmembrane receptors consisting of an alpha and a beta-chain, are known to provide physical linkages between the extracellular matrix and the actin cytoskeleton via adaptor proteins, e.g., talin, vinculin, paxillin, and *α*-actin [25]-[28], thus building a bridge between extracellular space and the cell’s interior [29],[30]. In addition, they initiate specific biochemical reactions that further regulate the formation of actin filaments locally, e.g., by influencing PIP2 and the cycling of Rho being GTP and GDP bound [21].

Our hypothesis is that transmembrane receptors like integrin might be affected mechanically by the micro-ranged geometry of the titanium surfaces, and successive biochemical spatial-temporal mechanisms regulate actin polymerization locally. To explore whether this could explain the observed actin filament patterns, i.e., on a planar surface the growth of long, roughly aligned stress fibers, i.e., filament bundles, and in cells on pillar structures only shorter filament segments on pillar tops and edges, we conduct a computational study focusing on the interplay of membrane related dynamics and actin formation.

### Related computational models

Many efforts have been made to study actin dynamics at a macroscopic level, incorporating actin’s three nucleotide forms (ATP-, ADP ·Pi-, ADP-bound), filament branching, capping and severing at different levels of detail [31]-[34]. For a comprehensive overview of experiments and models, exploring dynamics of filament formation and the regulation of formation- and branching-enabling proteins, see [19]. Most of the models are non-spatial.

The problem of representing spatial properties can be approached in different ways. For example, the rule-based modeling tool BioNetGen has been applied to model actin filaments growth [35], based on previous kinetic models of filament elongation, depolymerization [32] and branching [33]. The respective models allow studying length and branching structure of a single filament representing its structure as a graph, i.e., in terms of which molecule is bound to which, without positioning the actin filament in space. Microscopic simulations of actin filaments, i.e., where every molecule is represented by an individual model entity with its own position, include a Brownian dynamics simulation of ATP-actin polymerization [36],[37] with focus on the process of *treadmilling*, i.e., filament growth on the barbed end and simultaneous shrinking at the pointed end. The different polymerization schemes of lammellipodum and lammellum are analyzed in [38]. There, the competing roles of ADF/cofilin and tropomyosin lead to two compartments in the cell, one dominated by ADF/cofilin and closer to the leading edge, and another dominated by tropomyosin. As a result, with the distance to the leading edge, a steep increase of filament length could be observed.

In [34], a comprehensive spatial model incorporating many previous efforts (cf. [19]) is presented. Based on partial differential equations, it is used to investigate the effect of the presence of N-WASp (nuclear Wiskott-Aldrich syndrome protein; known to activate Arp2/3, which in turn mediates filament branching) at the leading edge of migrating cells. By using differential equations, filaments are not individually represented, but their presence and mean length can be determined from the concentrations of filamentous actin and of barbed ends.

In our particle-based approach, we decided to leave many details of actin polymerization and depolymerization processes aside. To shed some light on the mechanisms that drive the observed actin filaments patterns on the micro-structured surface, processes at the membrane have to be integrated. Integrin receptors and the forming of focal adhesion complexes have been subject to a series of models, e.g., of focal adhesion-related signaling with focus on RNA inference [39], or focusing on mechanical aspects [40]. In the above examples the simulation approaches [41],[42] range from non-spatial deterministic population-based modeling (ODEs in [31],[33],[34],[39]), discrete stochastic ([32],[35]), via mesoscopic (PDEs in [34]) to microscopic techniques (individuals with Brownian motion in [38], Browian dynamics in [36],[37]).

### Focus of this work

Whereas most actin models are aimed at analyzing physiological processes that drive actin polymerization and depolymerization, our goal is to understand the impact of a micro-topographic material structure. The focus of modeling turns towards spatial temporal processes close to the membrane. For this, we need to describe geometric structures in continuous space and their interaction with the cell. Thus, a spatial modeling approach shall be pursued [43].

As polymerization of actin filaments shall be described as concrete structures developing in continuous space, approaches that are based on discrete space, or assume no volumes associated with the key players (e.g., as adopted in [44]) do not appear suitable. The same hinders exploiting approaches based on partial differential equations (e.g., as in [34]). A definition as cellular automata [45] would constrain the spatial dynamics to the chosen grid granularity and shape. To represent structures emerging from moving molecules in the cell, we thus pursue a microscopic, individual-based approach with movement of molecules approximated by Brownian motion, where molecules cease moving when binding to form filaments. We use an adapted variant of ML-Space [46] that allows placement of molecules in relation to their binding partners.

Similarly as in [38], we keep our model of filament formation as abstract as possible in order to reduce both the number of unknown parameters and the number of simulated particles. Regarding the former, while kinetics of regulatory proteins have been explored previously, conversion of these macroscopic rates to microscopic rates for particle-based simulation would require knowledge of size and diffusion constants of the involved species. Regarding the latter, particle-based simulation is computationally expensive, so simulating a realistic amount of entities (i.e., approaching the number of those present in the cell) becomes infeasible. Unlike models that cover the actin dynamics at the leading edge during cell migration, in our study, the processes of interest take place at the center of the osteoblasts growing on the micro-topographies.

In the following sections, we first present wet-lab experiments done in addition to previously published ones [9]. These experiments motivated the modeling efforts and influenced the choices of abstraction in the model. In the next section, we describe our choice of modeling and simulation approach and introduce the key reactions of the model created here. The following section contains results of wet-lab and dry-lab experiments. We conclude with model extensions and wet-lab experiments planned in the future.

## Methods

### Wet-lab experiments

#### ***Titanium arrays***

For the experiments, defined micro-structured titanium arrays (single sizing 10 × 10mm) with a periodical cubic pillar geometry with a pillar dimension of 5 × 5 × 5 *μ*m (length × width × height) and a pitch width of 10 *μ*m (subsequently called P-5 × 5 × 5 or P-5 × 5) were used. These samples were fabricated by using deep reactive-ion etching technology (DRIE) (Center for Microtechnologies ZFM, Chemnitz, Germany) for defined micro-structuring of the titanium wafers. As respective control planar titanium wafers were used (subsequently called Ref). Qualitative analysis of the samples was made by using field-emission scanning electron microscopy (FE-SEM Supra 25; Carl Zeiss, Jena, Germany).

#### ***Cell culture***

Samples were washed in 70% ethanol for 15 min, rinsed in phosphate-buffered saline (PBS) (PAA Laboratories, Pasching, Austria) and then placed into 4-well NUNC dishes (Thermo Fisher Scientific, NUNC GmbH & Co. KG, Langenselbold, Germany). Afterwards human osteoblastic cells (MG-63, purchased from ATCC; No. CRL-1427) were seeded at a density of 3×10^4^ cells/array in Dulbecco’s modified Eagle medium (DMEM) (Invitrogen GmbH, Karlsruhe, Germany), containing 10% fetal calf serum (FCS) (PAA Laboratories, Pasching, Austria) and 1% gentamicin (Ratiopharm GmbH, Ulm, Germany) at 37°C in a humidified atmosphere with 5% CO _2_.

#### ***Cell morphology***

Cell morphology of MG-63 cells was visualized after 24 h cultivation time on the titanium arrays (see Figure 1 top) by using the field-emission scanning electron microscope FE-SEM Supra 25 (Carl Zeiss, Jena, Germany) without gold coating at a low acceleration voltage of 1 kV. Before examination of cell morphology, cells that were grown on the titanium arrays for 24 h were fixed with 2.5% glutaraldehyde (1 h, 4°C), dehydrated through a graded series of acetone (30% 5 min, 50% 5 min, 75% 10 min, 90% 15 min, 100% twice for 10 min) and dried in a critical point dryer (K 850, EMITECH, Taunusstein, Germany).

#### ***Microscopic analysis of the actin cytoskeleton***

MG-63 cells were cultured on the samples for 24 h and then fixed with 4% paraformaldehyde (PFA; 10 min, room temperature). Afterwards cells were washed twice with PBS, permeabilized with 0.1% Triton X-100 (10 min, room temperature) (Merck, Darmstadt, Germany). Afterwards cells were incubated with phalloidin-TRITC (diluted 1:10) (Sigma Aldrich Chemie GmbH, München, Germany) for 30 min in the dark at room temperature, washed again and embedded with a cover slip in mounting medium. Actin staining was investigated with an inverted confocal laser scanning microscope LSM 410 (Carl Zeiss, Jena, Germany) equipped with a helium/neon-ion laser (excitation: 543 nm) and a ZEISS 63x water immersion objective (C-Apochromat 63, 1.25 W/0.17).

#### ***Actin quantification via FilaQuant software***

The confocal images (512 × 512 pixels) were used for subsequent actin quantification via mathematical image processing by the FilaQuant software [9],[47]. They were automatically processed in three steps: preprocessing, where the main sources of errors based on image acquisition, i.e., noise and irregular background illumination, were reduced, followed by feature detection and quantification. The resulting parameters for the description of actin filament formation were as follows: total filament length, average filament length, maximum filament length and orientation dispersion. The parameter “orientation dispersion”, also known as angular deviation, describes the presence of a preferred orientation of the actin filaments. We here use normalized values ranging from 0%, implying exactly one preferred orientation, to 100%, indicating the maximum possible value determined by the method ([48], p. 28) and thus a uniform distribution of oriented length to total length ratio. For examining actin filament formation in MG-63 cells on the pillared (P-5 × 5 × 5) and planar reference titanium array (Ref), 30 cells per specimen were analyzed with FilaQuant.

#### ***Microscopic analysis of integrin receptor β 1***

Integrin *β *1 : MG-63 cells were cultured for 24 h on the titanium samples, then washed twice with PBS (+ Ca ^2+^ and Mg ^2+^) and incubated with mouse anti-human monoclonal antibody against the integrin *β *1 (CD29, 1:20) (Beckman Coulter) for 30 min. After washing with PBS, cells were incubated with AlexaFluor 488-labeled secondary goat anti-mouse IgG (1:300) (Molecular Probes) for 30 min in the dark, afterwards fixed with 4% PFA for 10 min and embedded. The formation of integrin adhesions was analyzed with the LSM 410 using the argon laser (excitation: 488 nm) (Carl Zeiss).

Activated *β *1 integrin via 9EG7: 9EG7 detects a specific epitope of the receptor – the ligand-bound conformation of *β *1 integrins [49]. MG-63 cells were cultured for 24 h (DMEM, + 10% FCS (Superior, Biochrom AG/Merck) on the titanium samples, washed with PBS and incubated with the primary antibody purified rat anti-mouse CD29, 9EG7 (1:40 in PBS; BD Pharmingen, BD Biosciences) for 60 min at room temperature. After washing with PBS, cells were incubated with the secondary antibody Alexa Fluor^®^; 488 goat anti-mouse IgG (1:100 in PBS, Life Technologies GmbH, Invitrogen) for 30 min in the dark. After washing again, cells were fixed with 4% PFA for 10 min at room temperature and embedded in Fluoroshield (Sigma-Aldrich Co. LLC). The activated integrins were analyzed with the LSM 780 using the argon laser (excitation: 488 nm) (Carl Zeiss).

#### ***Protein extraction***

After 24 h cultivation time on the titanium arrays, cells were harvested with 0.05% trypsin-0.02% EDTA and kept on ice, immediately. Cell extracts were prepared with Bio-Plex Cell Lysis Kit (Bio-Rad Laboratories, Hercules, USA) according to the manufacturer’s recommendations. The cell lysates were sonicated and centrifuged at 8,000 g for 2 min at 4°C. The protein in the supernatants were pooled together and stored at -80°C. Total soluble protein concentrations were estimated by Bradford protein assay [50] and verified by Coomassie staining [51] so that equal amounts of total cellular protein could be used for subsequent Western Blot analysis and quantification of the phosphorylation level of signaling proteins.

#### ***Western blot***

For Western Blot analysis total cellular protein lysates were separated by SDS-PAGE and blotted on PVDF membranes. After the protein transfer membranes were blocked with 5% skim milk in Tris-buffered saline (TBS) and washed six times in TBS. For protein detection primary antibodies anti-Rho A, anti-ROCK and anti-vinculin (Sigma Aldrich Co. LLc, US) were incubated overnight at 4°C followed by a labeling with a horseradish peroxidase-conjugated secondary antibody (Dako, Glostrup, Denmark) for 1 h at room temperature. Protein signals were visualized by using SuperSignal West Femto Chemiluminescent Substrate (Pierce Biotechnology, Rockford, USA) for detection of peroxidase activity from HRP-conjugated antibodies (Thermo Fisher Scientific Inc., Rockford, USA. Band intensity was analyzed densitometricly with the Molecular Imager ChemiDoc XRS and Image Lab 3.0.1 software (Bio-Rad Laboratories, Hercules, USA). Each protein detection was repeated at least three times with individual prepared cell lysates from independent passaged cells.

#### ***Statistical analysis***

Statistical analysis was performed using SPSS-software version 15.0 for Windows (SPSS Inc., Chicago, IL, USA): Kolmogorov-Smirnov test and unpaired samples t-test. Data were presented as a mean ± standard deviation (SD).

### *In silico* modeling

#### ***Modeling and simulation approach: ML-Space***

ML-Space is a model description language for spatial simulation. It is based on ML-Rules [52], a multi-level modeling language that allows describing dynamically nested systems. Both ML-Rules and ML-Space are rule-based approaches (see also [53],[54]). While ML-Space aims to facilitate modeling and simulation with discretized and continuous space, we here focus on the latter part where entities are modeled individually moving in continuous space (hence we will use the term particles synonymously).

SRSim [55] is another approach bringing together rule-based modeling and spatial simulation. Recently, also the rule-based language Kappa has been equipped with a coarse grained Brownian dynamics execution approach [56]. However, neither supports nesting of entities, which is a distinctive feature of ML-Space and will be used here to represent regions that can contain the same kinds of entities as their surroundings, but where these entities may behave differently.

In our approach, Brownian diffusion of particles is represented by position updates in continuous space in discrete time steps. For each particle, such a simulation step consists of 

1. determining a vector such that the particle moves a random distance drawn from $N(0,2D_{i}\Delta t)$ along each coordinate axis, where *D*_*i*_ is the particle’s associated diffusion constant and *Δt* the time since the last position update attempt. This approach is derived from Einstein-Smoluchowski equation and commonly used for particle-based simulation (e.g., [38],[56]). The average step length here is chosen to correspond to the diameter of the smallest particle (or a factor thereof) and the time steps *Δt* between position updates are chosen accordingly for each particle.

2. determining overlap with other particles after the move, i.e., after updating the position by the said vector. If this results in overlap with other particles (i.e., a collision), either 

(a) letting the particles react, i.e., finding an applicable reaction rule (see below) in the model and applying the associated changes, then resolving the collision by moving the updated particle slightly such that it does not overlap the other anymore, or

(b) in absence of applicable reactions, undo move and start again with the first step, unless this is already the *4th* such attempt (number customizable); then conclude that there is no space for the particle to move.

3. scheduling a new move event for this particle in *Δt*. Check for changes in first-order events, i.e., rates of first-order reactions for this particle, and reschedule appropriate event like in classical stochastic simulation (cf. [57]).

Reactions are formulated as rules consisting of a left side specifying initial reactants, a right hand side with changed reactants and products, if applicable, and finally a rate expression. The rules can describe time-triggered or collision-triggered events. Time triggered events (zeroth- or first-order reactions) affect at most one (previously existing) model entity (although they may entail the creation of new ones). Here, the rate expression relates to the propensity of the reaction to occur as in usual stochastic simulation, i.e., time intervals between occurences of a reaction with rate *r*_*j*_ follow an exponential distribution with parameter $\frac{1}{r_{j}}$. A reaction applicable to several particles is scheduled for each of them separately, as the particles are distinct entities due to their different positions. Stochastic race is used to determine who is first. (Note that in non-spatial stochastic simulation, reactions of order larger than one, like the example given, are also time-triggered, and parameter of the exponential distribution from which the next reaction time is drawn is adjusted for the concentration of available reactants in the given volume).

Second-order reactions can happen only between spatially close entities. We thus call them collision-triggered, since they are only applicable when the entities’ movement (i.e., diffusion, see above) has resulted in a collision. The reaction rate expression is to be interpreted as probability of the reaction happening once the collision has already occurred. These probabilities can be calculated based on macroscopic and microscopic rate constants, particles diffusion constants and sizes [58], however, the latter are often not known with reasonable accuracy (and may depend on organism and cell type).

Reactions of order three or higher must be broken down into several elementary reactions of lower order, as is customary in spatial simulation, with intermediate entities or entity states.

Collision-triggered reactions may consist of a smaller entity entering a larger one it collides with, e.g., a cellular compartment. We use this here to specify regions of the cell with possible contact to the structures on the underlying titanium surface. As mentioned, rules can be specified as being applicable only to entities nested in a given other, i.e., compartment or region.

Second-order reactions may also involve binding of entities, i.e. the participants stay together after the reaction. Dissolution of a bond again may be a time-triggered reaction or triggered by collision with another, non-bound entity. The unbound entity or entities can simply diffuse freely again starting from their current position (which may lead to prompt rebinding; cf. [59]). For all entities, the number of binding sites and angles between binding sites must be specified, and an entity binding to a previously free binding site of an entity with an already occupied site will always be placed such that the relative angle matches the specification, independent of its previous position, i.e., the angle from which it collided. This is used here, for example, to achieve straight actin filament segments by placing an free actin binding to a barbed end of a filamentous actin directly opposite (i.e., at a relative angle of 180°) the latter’s already present binding partner at the pointed end.

A demo software tool containing the ML-Space simulator is provided as Additional file 1.

#### ***Model abstractions***

Since we will primarily focus on the interface of the (structured) surface and the membrane of the cell growing on it, and since the cells ultimately are lying relatively flat on the surface (see also the cell morphology shown in Figure 1), we use a two-dimensional model to describe the system (although ML-Space supports 2D and 3D simulations). Our model thus covers a section of the cell near the surface and involves both entities that are actually mostly part of the membrane (e.g., receptor complexes) as well as those from the cytoplasm (e.g., free actin). To keep computational effort to a reasonable limit, our models only comprise a section of the center of the cell. We chose an area large enough to fit 3 × 3 pillars of 5 *μ*m width with an equally wide gap between them.

Even this area can fit a much higher number of entities (many million proteins at a diameter of 4–6 nm, for example) than can be simulated in reasonable time (usually a five-digit number of steps per second depending on machine and scenario). We thus needed to simulate fewer actin molecules than realistically present in the considered area, usually a few thousand per run. Since what is observed in microscopic images of filament formation are actually *bundles* of actin filaments, we chose the size of actin particles larger than it should be relative to the surface structures. The simulation of actin binding in silico can be thought of as representing the formation of several filaments at once. We chose the remaining particle size parameters in proportion to the protein sizes (measured in number of amino acids) for lack of authoritative information, and their diffusion constants inversely proportional to the size’s square roots.

#### ***Model components***

Key model components, actin and integrin molecules, are represented in ML-Space code like this:

The parts in parentheses define that actin and integrin (receptor complex) entities are represented by circular shapes of previously defined size (model constants or parameters) and diffuse according to a previously defined diffusion coefficient. The integrin model entities also have an attribute representing whether the entity is currently part of a focal adhesion complex or not. The part in angle brackets gives the binding sites and their angles relative to each other. The pointed end is for connecting to an existing filament and the barbed end is where filament growth can subsequently continue. Two binding sites for branching are given to allow for branches extending to either side of the filament chain (at an angle of 70° relative to the growth direction).

In our model, the focal adhesion complexis represented solely by the integrin entity that forms part of it (whose attribute “focal” has the value “yes”); further binding partners of the complex are not explicitly represented in the model. Its sole binding site will later be used to bind an actin to form the start of a filament.

A full model description including definitions of used constants, the branch-initiating species’ definition and branching reactions is provided as Additional file 2.

#### ***Modeling surface structure***

The model also explicitly includes the surface structures. As ML-Space supports hierarchical nesting, e.g., to represent a cell nucleus and proteins inside and outside of it, the extra-cellular surface structure can be represented the same way as a cellular compartment.

A specially treated attribute “boundary” has been added whose value “soft” indicates that this structure is not bounded by a membrane, which would require placing other model entities completely on either side of it. With a soft boundary the structure represents a “region” whose boundary can be overlapped (whether a particle belongs to the structure or the surrounding is then decided by the position of the particle’s center). Still, rules have to be specified indicating which particles can enter and leave the region and with which probability they do so when their center moves across the boundary. Attribute values of the moving entities may be changed in the process.

The example would express that integrins can enter the surface structure anytime (probability 1 after the @ symbol) and that each integrin that moves onto a surface structure is considered to immediately bind to an (excluded) surface-sensing agent and become part of a focal adhesion complex (attribute change on the right). This may not be a realistic assumption. It should also be considered that a complex of proteins would in reality diffuse more slowly and thus the diffusion attribute should be changed along the way. Moreover, slowing diffusion in distinct regions typically implies accumulation of the slowed down entities in that region [60], while at the same time the slowdown of activated integrin at the surface structure might facilitate the recruitment of cytosolic proteins (as indicated in [61] for slow binding kinetics) and might help clustering the focal adhesion complexes at the surface structure. The forming of the focal adhesion/integrin receptor complex can thus alternatively be modeled to happen on surface structures with a certain rate *rIntComplexFormation*.

The above also includes a reaction of a focal adhesion complex dissolving, resulting in a freely moving integrin. This shall only be allowed if the respective focal adhesion has no actin (filament) bound, hence the requirement of an unoccupied binding site on the rule’s left hand side.

#### ***Model reactions: filament growth***

Since we aim to reproduce the observed growth pattern with as simple a model as possible, we omit details unrelated to wet-lab observations like actin phosphorylation state, also because more states or attributes would introduce more yet unidentified model parameters and increase the risk of overfitting [62]. We start with a very simple model where free actin exists in only one state (ready to bind to a filament), filaments grow only at the barbed end and filament formation is dependent on an activated integrin receptor/focal adhesion complex.

Here, OCC (or OCCUPIED) indicates that something, no matter what, is bound at this binding site, where FREE specifies the opposite. The keyword *new* (or a different one that occurs exactly twice, e.g., “bind”) indicates a bond of the respective two entities via the respective binding sites, or establishing of a new bond if it occurs on the right hand side of a rule. All our “filaments”, even those consisting of only two molecules, are considered to be immobile, i.e., we do not model any filament movement.

#### ***Model reactions: filament severing***

We include in our model a species filling the role of severing actin filaments on collision at the impact site (i.e., the filamentous actin it collided with). This species, called cofilin, can be active or not (although actual cofilin proteins influence actin filaments in significantly more complex ways [32],[34],[63]; note that the actual cofilin is inactive when phosphorylated).

The first rule specifies that if active cofilin collides with an actin that has another entity bound at the pointed end, all bindings of the actin are released (potential branching sites omitted above) with given probability. As binding is a symmetric relationship, what is bound at the pointed and barbed end will be affected by this rule, too: the value of its respective binding site will be set to FREE as well. The second rule says that the remaining filament part starting with a free pointed end actin will be dissolve from the pointed end (if the specified rate *rFilDissolution* is > 0; in our simulations we used an infinite rate, i.e., the whole chain will be converted to free actins in the same time step, as the same rule will be applied successively to all actins in the remaining filament trunk).

Wet-lab results indicated that cofilin activity is (negatively) regulated by actors related to the integrin receptor complex. We integrated two different potential regulatory relations into our model.

First, we let cofilin be deactivated on every contact with the focal adhesion complex, and get reactivated on its own (i.e., by agents assumed to be constant and not explicitly in the model) with a given rate *rCofilinReactivation*.

Secondly, we represented the intermediate steps by an intermediate component called *CofReg*, which stands in for several potential regulatory proteins possibly in a multi-step cascade whose details, including kinetic parameters, i.e., reaction rates, are not known. Our *CofReg* deactivates cofilin and is itself activated at the focal adhesion complex. We let *CofReg* appear near the receptor complex and disappear with a certain rate. Alternatively, one could simulate a fixed amount of *CofReg* entities that are activated near the receptor and get deactivated on their own, like cofilin. Both variants should lead to the same patterns with respect to amounts and distribution of active *CofReg* during the simulation. The second alternative requires more simulation effort for the inactive *CofReg* not present otherwise (and could also lead to more molecular crowding effects due to molecules impeding each other’s movement more often, which is undesired here since our 2-dimensional approach already leads to occasional spurious blocking of particles).

By setting either *pCofDeactAtInt* or *rCofRegAppearance* to 0 one can then select the mechanism to be simulated.

#### ***Simulation and experiments***

We simulated models with differences in some mechanisms, e.g., regarding cofilin activity (holding active cofilin constant, having it deactivated as first-order reaction, cofilin deactivation by the integrin/focal adhesion complex or by an intermediate entity that is itself activated by the receptor complex). For lack of information on the amounts of proteins of each type and the reaction probabilities, we tested different values for crucial parameters.

We simulated our model with all possible combinations of values for key parameters given in Table 1. In addition, we also ran simulations with cofilin activity regulation directly at the integrin receptor complex (*pCofDeactAtInt *= 1), i.e., without any *CofReg* (*rCofRegAppearance *= 0). For collision-triggered reaction, a decrease in the probability of the reaction happening on each collision and an increase in the amount of a reactant by the same factor have opposite effects on the number of occurring reactions, which roughly cancel each other. Probabilities of collision-triggered reactions were thus set to 1 or near 1, unless otherwise noted.

**Table 1:** Key parameters in model simulations

**Parameter**	**Type**	**Values used**
Actin	Amount	250, 500 ^(1)^
Cofilin	Amount	400, 800
Integrin	Amount	100, 200
rCofRegAppearance	Reaction rate (1st order)	0.5, 2
rCofRegDisappearance	Reaction rate (1st order)	0.5, 2
Angle deviation	Distribution	$N(0,{7.5{}^{\circ}}^{2})$,
		$N(0,{15{}^{\circ}}^{2})$,
		$N(0,{30{}^{\circ}}^{2})$
Surface structure	(Qualitative)	planar surface,(groves), pillars

Values were chosen after multiple experiments with values outside the given ranges had lead to either behavior similar to those reached with a combination of the above values, or to behavior not matching the wet-lab observations (e.g., very short filaments, or very long filaments in both the planar and the micro-structured setting). Cofilin activity regulation via intermediate step *CofReg* has been assumed above, i.e., *pCofDeactAtInt ****= ***0. For actin, initial amounts are given. Additional actin was generated during the simulation (at a constant rate, such that there were 5000 – 6000 actin entities at the end of each simulation). Note that our actin chains are supposed to represent filament bundles and thus the proportion of actin to other actors, especially cofilin and integrin, need not correspond to realistic conditions.

The simulations were repeated several times, with a very small variance. Thus, in the following we will focus on one set of results. The simulation end time was the same for all runs (48 a.u.) and chosen from experience with previous tests such that no significant change in key model outcomes, especially average filament lengths, should be expected anymore.

ML-Space model interpreter and simulator are implemented in JAVA and integrated into the modeling and simulation framework JAMES II [64], which also served as experimental framework for most of the parameter scan experiments. Some optimization experiments were executed by using the simulation experiment specification language SESSL[65]. MATLAB[66] was also used for the evaluation of larger result sets.

## Results

### Wet-lab experiments

#### ***Cell morphology***

The morphology of MG-63 osteoblasts after 24 h cultivation time on the titanium arrays is shown in Figure 1. The SEM images show that on the planar reference (Ref), the cells are flattened and attached to the surface with their whole cell body. In contrast, on P-5 × 5 × 5 cells preferentially adhere to the surface plateaus, namely the top of the pillars, and only the filopods reach the bottom of the surface. Regarding cell shape, cells are more elongated on the micro-pillared surface than on the planar sample.

#### ***Quantification of actin filament formation***

Actin is the major component of the cellular cytoskeleton and is of elementary importance for diverse cellular processes that control morphological and physiological cellular traits. We investigated actin cytoskeleton architecture in dependence on defined surface topography with confocal laser scanning microscopy subsequently followed by the quantification of actin filament formation via the software FilaQuant. Confocal microscopy demonstrated that on the planar reference (Ref) actin is organized in a network of long and well-defined stress fibers typically spanning the entire cell body (Figure 1 bottom). In contrast, on P-5 × 5 × 5 actin only forms short fibers, which are concentrated on the edges and tops of the micro-pillars and demonstrate an adaptation of actin filament formation to the underlying surface topography [8]. Based on the confocal images, actin filament formation was subsequently quantified automatically via FilaQuant software [9],[47],[48]. The average length of filaments (*μ*m) was lower by more than 60% on the pillared structure in comparison to the planar structure, the spatial orientation was significantly disturbed as calculated by the parameter orientation dispersion [9].

#### ***Protein expression***

**Figure 3:**
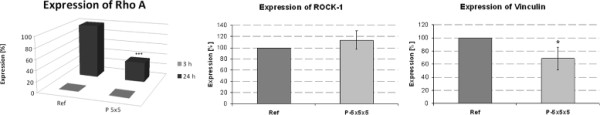
**Densitometric analysis of the protein expression of Rho A, ROCK-1 and vinculin in MG-63 osteoblasts after 3 h (Rho A only) and 24 h.** On the micro-pillared surface (P-5 × 5 × 5), Rho A expression is significantly lower compared to the planar reference (Ref). Vinculin expression is also lowered, ROCK-1 expression changes are not significant. (Mean ± SD, unpaired t-Test, *n *= 6 for Rho A, *n *= 3 for ROCK-1, *** *p *< 0.001) (Molecular Imager^®^; ChemiDoc™ XRS, Quantity One^®^; 1-D analysis software, Bio-Rad).

The actin-binding proteins Rho A and ROCK-1, also linked with cofilin, are of essential relevance in actin remodeling process. Western blot analysis revealed that the expression of Rho A in MG-63 osteoblasts on P-5 × 5 × 5 was significantly lower after 24 h compared to the reference (Ref; Figure 3). Also vinculin as protein in focal adhesions was significantly lower expressed in cells on pillars. In contrast no statistical difference in ROCK-1 protein expression between the micro-pillared and the planar titanium surface could be detected.

#### ***Formation of β 1 integrin adhesions***

**Figure 4:**
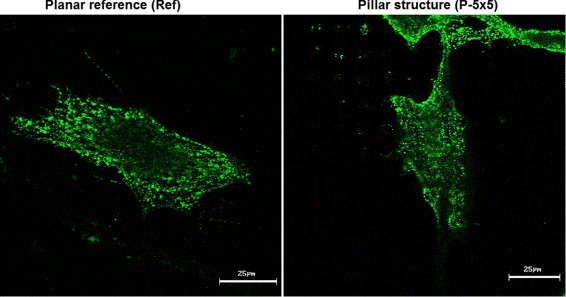
**Localization of *β *1 integrin in MG-63 osteoblasts on the planar surface (left) and the micro-pillars (dimension 5 × 5 × 5 *μ*m, right) after 24 h.** On both titanium surfaces there is a homogeneous distribution of integrin *β*1 receptor throughout the whole cell (LSM 410, Carl Zeiss).

Integrins are central members of focal adhesions sites between cells and biomaterial and play a major role in receptor-mediated adhesion processes. Via distinct adaptor proteins they are linked to the actin cytoskeleton and thus have decisive effects on its formation. Confocal microscopic analysis of *β *1 integrin formation in MG-63 cells after 24 h showed that on both titanium surfaces – the micro-pillared P-5 × 5 × 5 surface as well as on the planar reference (Ref) – there is a homogeneous distribution and no clustering or accumulation of *β *1 integrins near micro-pillars could be observed (Figure 4).

**Figure 5:**
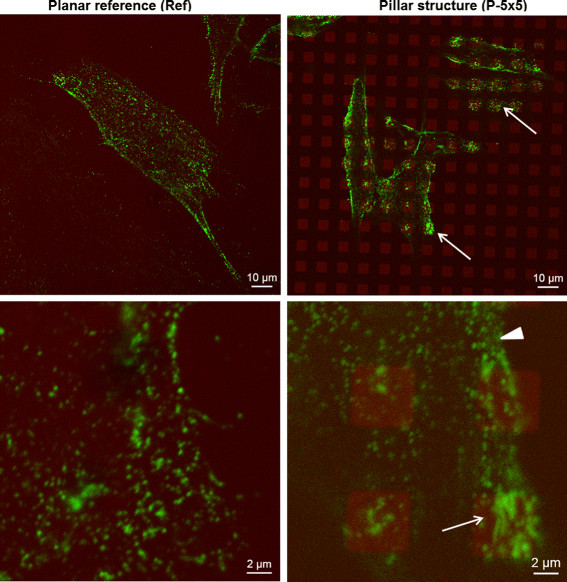
**Activated *β *1 integrin, detected by 9EG7, in MG-63 osteoblasts on micro-pillars.** On the planar surface (left) activated *β *1 integrins are homogeneously distributed, whereas on the micro-pillared surface (right) activated *β *1 integrins are partly clustered on top of the pillars (arrows). Note that activated *β *1 integrins are not clustered solely on pillars but formed also aggregates in parts in between the pillars (arrowhead). Confocal microscopy (LSM 780, Carl Zeiss, bars = 10 *μ*m upper row, 2 *μ*m lower row).

However, these measurements cover integrins in any state, not only activated integrins required for formation of focal adhesions. More recent experiments, also motivated by the results of our simulation experiments, investigate the activated integrin state via the 9EG7 antibody. They revealed that on pillars the activated integrins of the cells seem to be more clustered in parts compared to the surroundings (Figure 5). However, activated integrins can be still found between the pillars (Figure 5 bottom right), whereas the actin appears strictly constrained to the pillared regions. Note that vinculin, which is also part of focal adhesions, was already previously found to be clustered on pillars ([8], Figure ten).

### Simulation

In our *in silico* experiments, we compare different strategies of activating integrin, analyze the impact of changing the amount of integrin, actin, or cofilin in the system, and take a closer look at the impact of the severing mechanisms and the local distribution of the severing agent.

#### ***Amounts of key players***

The amount of *actin* is the most obvious determining factor of filament lengths. Too few of them, and filaments eventually run out of free monomers to bind, staying short. Too many actins, however, and many simulation steps consist of actin moves and possibly collisions without reaction, especially at the beginning of the simulation when few nucleation points or filament ends are available. Since actins in filaments are more tightly packed than free actins, once enough actins are bound to filaments, the few left free actins (or those freed again by depolymerization) move through larger patches of empty space before occasionally encountering a filament to bind to again. Thus, we decided to start with a comparatively low amount of actin and to create actin molecules during the simulation to roughly maintain the density of free actin. This can be interpreted as recruitment of free actin from the cytosol. Once we did this, the initial amount of actin was no longer a key parameter for the length of filaments encountered later.

**Figure 6:**
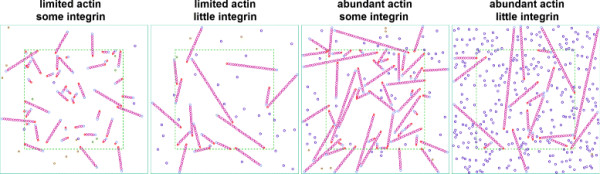
**Simulation illustration with small single surface structure with a fixed amount of actin and no limitation of filament orientation or severing (i.e., no cofilin).** Magenta circles represent actin entities with other entities bound at both ends (i.e., in filament chains), light blue circles are actins at filement ends (i.e., occupied pointed end, free barbed end), Purple/dark blue circles: free actin, smaller red circles: integrins at start of filament, dark green and brown circles, free integrin on and outside of a surface structure. Surface structure boundaries marked by green dashed lines. Too little actin can lead to relatively short filaments (left), too little integrin and abundant actin to a few long filaments and many free actins that have no room to bind to a filament (right). Filament “growth” can also be impeded by the two-dimensional approach where filament crossing or bending is not allowed (center right; note also several free actins trapped in regions bound by different filament segments).

Since in our model filament formation starts at an *integrin* receptor complex and we allow only one filament at each of them, the amount of integrins in the system limits the number of filaments that can form. When integrins could easily turn into focal adhesions (and thus filament nucleation points), their amount was indeed found to negatively influence the resulting average filament length (as illustrated in Figure 6), at least when keeping other parameters constant. This means that when actin is added at a constant rate as above and filaments are severed, a simulation with more integrin must be run longer to exhibit filaments of same average length as a simulation with fewer integrin. When filament severing happens often, the integrin amount becomes less relevant: filaments stay comparatively short independent of whether there are few or many of them.

**Figure 7:**
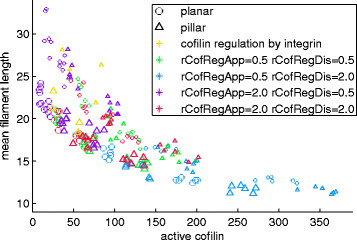
**Relation of filament size to amounts of active cofilin and other parameters.** Each marker indicates the result of a simulation run with different parameters. Colors denote parameters related to the cofilin activation mechanism, marker types distinguish simulations of pillar structure systems (triangles) and planar surfaces (circles), and larger markers indicate simulations with 200 initial integrin entities vs. 100 for the smaller markers (mostly to be found above the former). More active cofilin thus coincides with shorter filaments.

More *cofilin*, i.e., filament severing agent, in the simulation expectedly lead to shorter filaments (again when keeping other parameters constant). However, it was the amount of *active cofilin* that is relevant for filament lengths, and the amount of active cofilin depends on these other parameters, i.e., the rates of reactions regulating cofilin activity (see “Severing by cofilin” below and Figure 7), so the total cofilin amount alone is not a key parameter.

#### ***Mechanisms of integrin activation***

The mechanism by which free integrin turned into focal adhesions – with a certain stochastic rate (but only when on surface structures) or instantly upon entering such a surface structure (cf. “Modeling surface structure”) – made no difference to the simulation results. In the former case, increasing this rate and increasing the amount of integrin had the same effect – what mattered was the amount of active integrin/focal adhesions available for filament formation.

The initially hypothesized slowdown of integrin on pillars in our simulations leads to accumulation on these structures. When at the same time integrins cannot easily enter a surface structure (see also Additional file 3), a rougly homogeneous distribution of integrin can be achieved, too (cf. Figure 4). However, in our simulation most integrins eventually organize in focal adhesions. This is partly because we limited the number of integrins to reduce calculation time. Nevertheless, the wet-lab observation of more activated integrin on pillars than between them can thus also arise in models without a slowdown.

#### ***Severing by cofilin***

We compared cofilin regulation directly at (i.e., on collision with) the focal adhesion complex with a short cascade with an intermediate signaling entity, *CofReg*. The amount of active cofilin in the whole system depends on the parameters of the cofilin-regulating reactions (and the total amount of cofilin). With more *CofReg* (*rCofRegAppearance > rCofRegDisappearance*, purple markers in Figure 7), only a small fraction is active and filaments grow longer on average, with little *CofReg* (*rCofRegAppearance < rCofRegDisappearance*, blue markers), filaments are severed earlier and/or more often.

**Figure 8:**
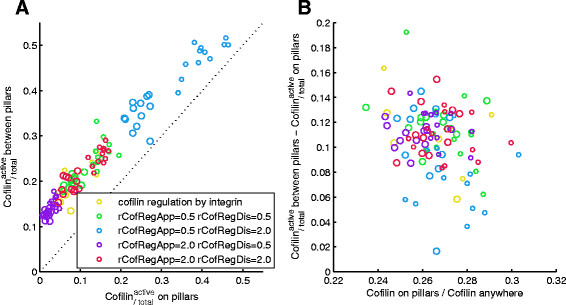
**Overrepresentation of active cofilin between pillars due to (de-)activation mechanism tied to the focal adhesion complex.** (Rate parameter names related to CofReg, *rCofRegAppearance* and *rCofRegDissapearance* slightly shortened). **A**: The fraction of cofilin that is active is higher in the region between surface structures than on surface structures (all points are above the dotted line, where both would be equal). **B**: The fraction of cofilin that is located on pillars (x-axis) is often slightly lower than the pillars’ share of the system size (29.7%), but unrelated to the difference in activity ratios of cofilin between the respective regions (note that each marker’s position on the y-axis corresponds to the respective markers’ distance from the dotted line in the left panel).

Since we assume that a significant part of the cofilin regulation mechanism happens in regions where the focal adhesion complexes reside, we compared the ratio of active cofilin to total cofilin in the respective regions, i.e., on and between surface structures (pillars). A small difference can be seen between the respective fraction for surface structures and for regions between them, also when cofilin (de)activation does not depend on an intermediate signaling entity (see Figure 8A; note that the comparison only makes sense for experiments with pillared surface structures, not for planar surfaces). This can go so far that there is almost no active cofilin on pillars, but at the same time the vast majority of cofilin between pillars is also inactive. This is not an artifact of a skewed overall distribution of cofilin (e.g., resulting from accumulation outside pillar regions; Figure 8B).

Note that all shown results stem from simulations with the same rate at which deactivated cofilin becomes active again (*rCofilinReactivation*). With higher or lower values for this rate, the active cofilin ratios can be made higher (towards the blue markers in Figure 8A) or lower (towards the purple markers), respectively.

**Figure 9:**
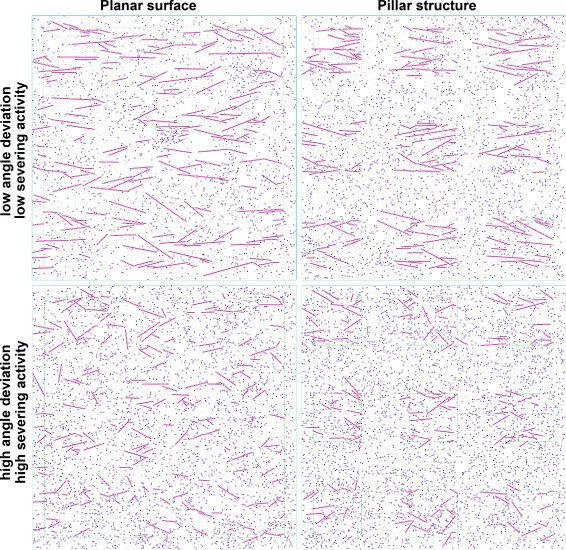
**Simulation results with different severing agent amounts and orientation dispersion values.** See also Tables 2 and 3 and note that the difference in conditions between upper left and lower right reflects wet-lab observations.

Also, the pillared region experiments lead to slightly shorter actin filaments than comparable experiments with a planar surface (Figure 9 right vs. left; cf. Tables 2 and 3). As the ratio of active cofilin vs. total cofilin on pillars is roughly equal to the one in comparable planar surface simulations, we attribute the shorter filaments in pillar structure simulations to the higher chance of filaments being cut off between pillars rather than on them.

**Table 2:** **Parameters and results of simulation runs shown in Figure **9

**Figure **9** panel**		**Top left**	**Top right**	**Bottom left**	**Bottom right**
rCofRegAppearance		2.0	2.0	0.5	0.5
rCofRegDisappearance		0.5	0.5	2.0	2.0
Angle deviation		$N\left(0,{15^{\circ }}^{2}\right)$	$N\left(0,{15{}^{\circ}}^{2}\right)$	$N\left(0,{30{}^{\circ}}^{2}\right)$	$N\left(0,{30{}^{\circ}}^{2}\right)$
Cofilin${}_{/\text{total}}^{\text{active}}$	on pillars	0.028	0.021	0.22	0.26
	between pillars		0.141		0.38
Average filament length (# particles)		21.1	18.5	13.0	11.4
Maximum filament length (# particles)		69	58	29	33

All four runs had these parameter values in common: initial actin amount 500 (changes here did not significantly change the results as most actin was “produced” during the simulation), integrin amount 200 (a lower amount, 100, lead to 20% longer filaments in situations where cofilin activity was low, ie corresponding to the top panels), cofilin amount 800 (lower values lead slightly longer filaments in *all* four settings).

**Table 3:** Quantification of wet-lab and simulation experiments

	***in vitro *** [9]		***in silico* (cf. Figure **9)			
			**low angle deviation**		**high angle deviation**	
			**low severing**		**high severing**	
	**Ref**	**P-5 × 5 × 5**	**planar**	**pillar**	**planar**	**pillar**
Average filament						
length *μ*m)	9.7 ±1.5	3.1 ±1.5	**1.09 ±.03**	0.90 ±.03	0.64 ±.03	**0.55 ±.03**
Maximum filament						
length (*μ*m)	51.5 ±11.9	6.7 ±2.0	**3.03 ±.33**	2.86 ±.17	1.82 ±.35	**1.77 ±.18**
Orientation						
dispersion (%)	66 ±14	84 ±10	**36 ±1**	36 ±1	65 ±1	**65 ±1**

Ref and P-5 × 5 × 5 refer to wet-lab experiments for planar and pillared surfaces (taken from ([9], Table one); *p****<***0.001 for the changes in all three quantified properties; averages and standard deviation of 30 cells per specimen). The final four columns refer to the simulation experiments (averages and standard deviation of seven simulation runs for each parameter combination; filament length calculated based on an actin diameter of 0.05 *μ*m). The two columns in bold there best reflect the changes of conditions for the actual cell. Changes in average filament length significant at *p****<***0.001 planar vs. pillar with same severing agent amount and angle deviation, all changes significant when comparing the first and last experiment column. Note that length values *in vitro* and *in silico* are not comparable – the FilaQuant software processing florescence microscopy images has cutoff parameters (regarding length and thickness of lines to consider a filament) while for the simulation every integrin/focal adhesion with at least one bound actin is considered a filament.

#### ***Filament orientation and branching***

Average filament length is slightly negatively correlated with our parameter angular deviation, which lets the filaments grow in similar direction, again when holding other parameters constant. More specifically, here the angle of each filament relative to the horizontal is set to a normally distributed value with mean 0 and a certain standard deviation (representing orientation dispersion). When filaments are more closely aligned, they can become slightly longer on average, mostly because when we allow filaments to grow in whichever direction they please (which would be the direction from which the first bound actin approached the original focal integrin), they get in each other’s way more often, limiting further growth (provided the other chosen parameters allow long filaments in principle). This could be considered an artifact of the chosen two-dimensional approach, assuming that one filament could simply grow above or below the other for a small segment in a three-dimensional approach.

**Figure 10:**
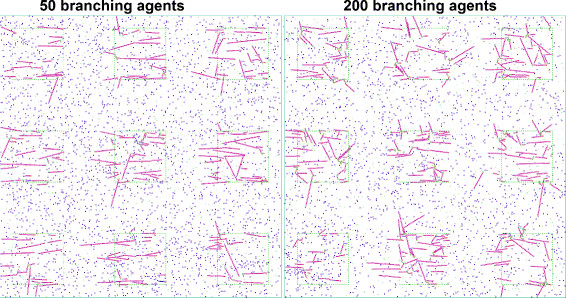
**Simulation results with branching enabled.** Other model parameters correspond to the low orientation dispersion, low severing situation (Figure 9 top). The amount of integrin (and thus maximum number of filaments) was 200.

Occurrences of one filament segment preventing further growth of another one are slightly more frequent in simulations where proteins are allowed to branch, i.e., where a certain amount of Arp2/3 entities are available from the simulation start. Here, overall filament sizes (when counting all entities in one filament complex, i.e., all branches of it) become larger relative to simulations with otherwise equal parameters but only straight filaments, although the longest segment chains in filaments seem shorter on average (cf. Figure 10). The angular deviation model parameter then has little effect on the orientation dispersion observed in the simulation, as branches always grow with an angle of 70° relative to the filament from which they branch off, leading to differently oriented branches.

## Discussion

So far we focussed on the effect of changes to single or few parameters while keeping the others constant. We also found that the “structure parameter”, i.e., the size of the area where the cell has surface contact and thus where filament growth can start and where the actin depolymerizing factor (cofilin) is regulated, can already explain part of the lower filament length in systems with the pillar structure compared to planar surfaces (Figure 9 left vs right).

Wet-lab experiments demonstrated that several of the aforementioned parameters (e.g., orientation dispersion and severing agent regulation as exemplified by Rho and vinculin expression) are different between the two situations, it is more appropriate to compare the planar surface simulations with low dispersion and low severing with the pillar structure simulation with high orientation dispersion and high severing agent (cofilin) activity (Figure 9 top left vs. bottom right). Then, the difference in filament lengths and patterns becomes more pronounced (see also Table 3).

Since we could establish that average filament lengths are sensitive to several parameters, some of which should be different between cells on planar surfaces and those on micro-structured ones, tweaking these parameters in one of the two settings can make the difference between their simulations larger or smaller.

## Conclusion

Our results indicate that sensing mechanisms and bio-chemical regulation of actin filament severing via cofilin might play a central role in explaining the phenotypical differences between osteoblasts grown on planar vs. geometrically micro-structured surfaces, the former due to the apparent concentration of filaments in areas where the cells had surface contact, the latter because of expression differences in regulatory proteins upstream of cofilin.

Based on the wet-lab results, we developed a spatial computational model of actin filament formation with several abstractions that lumped multi-step processes with yet unquantified components into single steps. First, based on a hampered entry of integrin into the pillar structure regions and a subsequent slowdown of integrins, the observation of homogeneously distributed integrin in wet-lab experiments could be reproduced. Subsequent wet-lab experiments then showed that activated integrins are slightly clustered on pillars. In earlier studies, it had already been shown that vinculin can be found strongly clustered on pillars. From both finding, we hypothesized that the focal adhesions, of which vinculin is a part, indeed form predominantly on pillars.

The spatial patterns of further selected members of the focal adhesion complex will be analyzed in future studies, also to shed light on their role in regulating the actin cytoskeleton. Our results indicate that filament growth pattern can result from the receptor complex’ role in regulating the actin depolymerizing factor (ADF)/cofilin.

Due to deactivation of cofilin in the vicinity of focal adhesion complexes (containing vinculin and activated integrin), which were clustered on the pillars, a higher concentration of active cofilin could be found between the pillars in our simulations, which increased the probability of actin filaments being cut off outside the pillar structures.

The resulting differences in our simulations, if we assume otherwise identical model parameters (i.e., reaction rates and actor amounts) for structured and non-structured surfaces, appear less pronounced than in *in vitro* observations. However, our findings in wet-lab experiments suggest that these identical model parameters are not realistic. e.g., we find a higher alignment (i.e., orientation dispersion) of actin filaments and higher Rho expressions on planar surfaces. The simulation results for pillar structure surfaces and those for planar surfaces were even closer to the wet-lab observation if these measured differences were added explicitly to the simulated models, i.e., orientation dispersion and the regulation parameter for cofilin were adjusted accordingly. The effect of shorter actin filaments on pillared structures compared to planar ones is, however, still more prominent *in vitro* than observed *in silico*. Therefore, current wet-lab studies are aimed at revealing possible mechanisms behind, among other things, the differences in orientation dispersion and processes that could induce the release and activation of additional cofilin at the pillared surface.

Further potential expansions of our approach include wet-lab experiments to measure concentrations of proteins that were lumped together into one model entity here, and the identification of realistic amounts and rate parameters for the reactions included in the model. First experiments indicate that, for example, the concentration of actin is significantly lower in the pillared case, although it remains to be seen whether this is the cause or an indirect effect of filaments not being able to grow long. Also the question what lies behind the observed phenomena of different actin alignments and species concentrations like Rho needs further exploration.

On the computational side, the rule-based spatial modeling approach ML-Space allowed to probe different hypothesis and a successive extension of the model easily. In addition to the particles, their location, spatial extension, Brownian movement and reaction, the model explicitly includes the surface structures by exploiting the hierarchical nesting in ML-Space. The approach can be expanded to allow for movement of bound entities together, i.e., filaments, instead of fixing their position in the event of binding, to find ways of representing the cellular stress to be the source of orientation alignment, and to speed up the simulation to allow more simulation runs with more realistic protein sizes and consequently many more proteins in the system.

## Competing interests

The authors declare that they have no competing interests.

## Authors’ contributions

AB and AU developed the model, AB executed the in-silico experiments, BN, CM and SS designed the wet-lab experiments, CM and SS executed the wet-lab experiments, AB, CM, BN and AU wrote the paper. All authors read and approved the final manuscript.

## Supplementary Material

Additional file 1 **Simple Simulator with command-line interface.** Zip archive containing four files: the simulator software (.jar file), the example model (.mls file), and.bat and.sh files for execution on Windows or Linux machines, respectively. A Java Runtime Environment (version 7 or higher) is required. By default, the example model will be simulated for 48 units of time, image files of the simulation state (cf. Figure 9) at start and end of simulation as well as several intermediate time points will be written to the current directory and two csv files will be created, one for complex size (i.e., filament length) data and one for key entity amounts at selected simulation time points. Click here for file

Additional file 2 **Complete model specification in ML-Space.** Parts highlighted in green are comments. The model consists of four parts: definition of constants, e.g., for reaction rates, entity properties (size, diffusion) or system size (lines 1–69), definition of species (lines 70–80), initial state definition (here consisting of three parts of which only one is used, to allow switching between simulation of planar surfaces, micro-pillared structures or groves; lines 81–111) and finally the rules. A plain-text equivalent of the model (i.e., without line numbers and syntax highlighting) is included in Additional file 1. Click here for file

Additional file 3 Model of hampered movement onto structures and slowing down. Click here for file
