# Supplementary material for: Membrane related dynamics and the formation of actin in cells growing on micro-topographies: a spatial computational model
Source: BMC Syst Biol. 2014 Sep 9;8:106. doi: 10.1186/s12918-014-0106-2 (PMC4363941; doi:10.1186/s12918-014-0106-2)
Supplement: Additional file 2 — Complete model specification in ML-Space. Parts highlighted in green are comments. The model consists of four parts: definition of constants, e.g., for reaction rates, entity properties (size, diffusion) or system size (lines 1–69), definition of species (lines 70–80), initial state definition (here consisting of three parts of which only one is used, to allow switching between simulation of planar surfaces, micro-pillared structures or groves; lines 81–111) and finally the rules. A plain-text equivalent of the model (i.e., without line numbers and syntax highlighting) is included in Additional file 1. [file s12918-014-0106-2-S2.pdf]

```
// First part: definition of constants used later in species and reaction definitions:
// reaction probabilities/rates
filFormProb := 1.;
pActBind := 1.;
pActRelease := .3; // probability of cofilin hitting filament leading to dissolution
rFilDissolution := Infinity;
rActinAppearance := 1;
pCofDeact := 0.9; // probability of Cofilin deactivation on collision with regulating entity
rCofReact := 0.1; // rate of cofilin reactivation (no external trigger)
rIntActivation := 9.;
rIntComplexDis := 3;

skipCofRegSwitch := 0;
cofRegProdMechanismSwitch := 1; // boolean parameter:
// CofReg always in System, activated on contact with Integrin, deactivated stochastically anywhere,
// or CofReg appearing near Integrin, disappearing stochastically anywhere
rCofRegAppearance := 0.5 * cofRegProdMechanismSwitch * skipCofRegSwitch;
rCofRegDissappearance := 2. / rCofRegAppearance * cofRegProdMechanismSwitch*skipCofRegSwitch;
rCofRegDeact := 1 * (1-cofRegProdMechanismSwitch) * skipCofRegSwitch;
pCofRegAct := 1 * (1-cofRegProdMechanismSwitch) * skipCofRegSwitch;
pCofDeactAtInt := pCofDeact * skipCofRegSwitch;

// species size/movement constants
actinDiam := 0.01;
actinBaseSize := PI*actinDiam^2;
actinDiff := 0.3;
// size of further species determined by number of amino acids compared to actin's 377
integrinSize := actinBaseSize / 377 * 988; // 788-1188
integrinDiff := 0.05;
cofSize := actinBaseSize / 377 * 166;
cofDiff := 0.6;
arpDiff := actinDiff;
arpSize := actinBaseSize / 377 * 406; // 394-418
cofRegDiff := (actinDiff + cofDiff) / 2;
cofRegSize := (actinBaseSize + cofSize) / 2;
actinScaling := 4; // oversized actin s.t. chains in simulation represent filament bundles
actinSize := actinBaseSize * actinScaling; // for separate overriding

// initial amounts
totalActin := 1000; // total; not all initially on surface structure
totalIntegrin := 200;
totalCofilin := 400;
totalArp := 50; // branch-enabling entity
totalCofReg := 200; // actual number dependent on switches, see next line
numCofReg := totalCofReg * (1-cofRegProdMechanismSwitch) * skipCofRegSwitch;

// system size properties
shortSize := 2;
longSize := 10;
gapSize := 0.5;
sysSize := (longSize+2*gapSize)^2;

// system surface structure properties
surfStructSwitch := 1; // 0: flat, 1: pillars, 2: groves
sysFlat := [1-surfStructSwitch / 2];
sysPillar := 1-(surfStructSwitch-1)^2;
sysGrove := [surfStructSwitch / 2];
pillarNum := 9;
idxP := ((pillarNum^0.5)-1)/2;
ridgeNum := 3;
idxR := (ridgeNum-1)/2;

```

```
// information for simulator:
postponedRegionInit := 1; // simulator switch for distributing entities
// initially defined outside of pillar regions between _and_ on them
initialAbsoluteAngle := 0; // horizontal default filament orientation
angleDevDeg := 30; // specified in degrees for easier overriding
initialAbsoluteAngleDeviation := angleDevDeg*pi/180; // degree->radians conversion

// species definitions:
SurfStruct(shape:rectangle,aspectratio:(1,1),boundary:soft,size:[0...sysSize]);
System(shape:square,size:sysSize);

Actin(shape:circle,size:actinSize,diffusion:[0...actinDiff])
     <pointed:0,barbed:180*pi/180,branch1:110*pi/180,branch2:250*pi/180>; // always bind-ready
Integrin(shape:circle,size:integrinSize,diffusion:[0...integrinDiff],focal:{yes,no})<bs:0>; // active only
Arp23s(shape:circle,size:arpSize,diffusion:[0...arpDiff])<fil:0,straight:180*pi/180>; // for side branching
Cofilin(shape:circle,size:cofSize,diffusion:cofDiff,active:{yes,no});
CofReg(shape:circle,size:cofRegSize,diffusion:cofRegDiff,active:{yes,no});


// initial state definition
// the constants sysPillar, sysFlat and sysGrove are defined above (i.e. derived from "surfStructSwitch")
// such that two of them are 0 and one of them is 1 (i.e. only one of the three blocks below is relevant)
0+sysPillar System(position:(0,0))[
    totalActin Actin(diffusion:actinDiff) +
    totalCofilin Cofilin(active:yes) +
    totalIntegrin Integrin(diffusion:integrinDiff,focal:no) +
    totalArp Arp23s(diffusion:arpDiff) +
    FOR x:=-idxP:idxP {
      FOR y:=-idxP:idxP {
        1 SurfStruct(size:shortSize,relpos:(x*2*shortSize,y*2*shortSize))
      }
  }]

 +sysFlat System(position:(0,0))[
    totalActin Actin(diffusion:actinDiff) +
    totalCofilin Cofilin(active:yes) +
    totalIntegrin Integrin(diffusion:integrinDiff,focal:no) +
    totalArp Arp23s(diffusion:arpDiff) +
    1 SurfStruct(size:longSize,relpos:(0,0))
]

 +sysGrove System(position:(0,0))[
    totalActin Actin(diffusion:actinDiff) +
    totalCofilin Cofilin(active:yes) +
    totalIntegrin Integrin(diffusion:integrinDiff,focal:no) +
    totalArp Arp23s(diffusion:arpDiff) +
    FOR y:=-idxR:idxR {
      1 SurfStruct(size:shortSize*longSize,aspectratio:(longSize,shortSize),relpos:(0,y*2*shortSize))
}];


// transfer rules: unimpeded migration onto/off surface structures
Actin() + SurfStruct() -> SurfStruct()[Actin()] @ 1
SurfStruct()[Actin()] -> Actin() + SurfStruct() @ 1
Cofilin() + SurfStruct() -> SurfStruct()[Cofilin()] @ 1
SurfStruct()[Cofilin()] -> Cofilin() + SurfStruct() @ 1
Integrin() + SurfStruct() -> SurfStruct()[Integrin()] @ 1
SurfStruct()[Integrin()] -> Integrin() + SurfStruct() @ 1
Arp23s() + SurfStruct() -> SurfStruct()[Arp23s()] @ 1
SurfStruct()[Arp23s()] -> Arp23s() + SurfStruct() @ 1
SurfStruct()[Integrin(focal:no)] -> SurfStruct()[Integrin(focal:yes,diffusion:0)] @ rIntActivation
Integrin(focal:yes)<bs:free> -> Integrin(focal:no,diffusion:integrinDiff) @ rIntComplexDis
```

```
// filament formation
Actin()<pointed:free> + Integrin(focal:yes)<bs:free>
   -> Actin(diffusion:0)<pointed:bind>.Integrin(diffusion:0)<bs:bind> @ pActBind
Actin()<pointed:free> + Actin()<pointed:occ,barbed:free>
   -> Actin(diffusion:0)<pointed:bind>.Actin()<barbed:bind> @ filFormProb

// Cofilin and CofReg activity regulation
Integrin(focal:yes) -> Integrin() + CofReg(active:yes) @ rCofRegAppearance
CofReg() -> @ rCofRegDissappearance
SurfStruct()[Integrin(focal:yes) + CofReg(active:no)] -> SurfStruct()[Integrin() + CofReg(active:yes)] @
     pCofRegAct
CofReg(active:yes) -> CofReg(active:no) @ rCofRegDeact

Integrin(focal:yes) + Cofilin(active:yes) -> Integrin() + Cofilin(active:no) @ pCofDeactAtInt

CofReg(active:yes) + Cofilin(active:yes) -> CofReg() + Cofilin(active:no) @ pCofDeact
Cofilin(active:no) -> Cofilin(active:yes) @ rCofReact

// filament destruction
Cofilin(active:yes) + Actin()<pointed:occ>
   -> Cofilin() + Actin(diffusion:actinDiff)<pointed:release,barbed:release> @ pActRelease
Actin()<pointed:free,barbed:occ> -> Actin(diffusion:actinDiff)<barbed:release> @rFilDissolution
// cutoff filament becoming mobile again
Actin(diffusion:0)<pointed:free, barbed:free> -> Actin(diffusion:actinDiff) @ Infinity
// Integrin(diffusion:0)<bs:free> -> Integrin(diffusion:integrinDiff) @ Infinity
// not if integrin complex is immobile itself, see above

// actin creation
System()[] -> System()[Actin(diffusion:actinDiff)] @ rActinAppearance
SurfStruct()[] -> SurfStruct()[Actin(diffusion:actinDiff)] @ rActinAppearance

// side branching
Arp23s()<fil:free> + Actin()<pointed:occ,branch1:free,branch2:free>
    -> Arp23s(diffusion:0)<fil:bind>.Actin()<branch1:bind> @ 1
Arp23s()<fil:free> + Actin()<pointed:occ,branch1:free,branch2:free>
    -> Arp23s(diffusion:0)<fil:bind>.Actin()<branch2:bind> @ 1
Actin()<pointed:free> + Arp23s()<fil:occ,straight:free>
    -> Actin(diffusion:0)<pointed:bind>.Arp23s()<straight:bind> @ 1
Arp23s(diffusion:0)<fil:free,straight:free> -> Arp23s(diffusion:arpDiff) @ Infinity
```
