# Supplementary material for: Membrane related dynamics and the formation of actin in cells growing on micro-topographies: a spatial computational model
Source: BMC Syst Biol. 2014 Sep 9;8:106. doi: 10.1186/s12918-014-0106-2 (PMC4363941; doi:10.1186/s12918-014-0106-2)
Supplement: Additional file 3 — Model of hampered movement onto structures and slowing down. [file s12918-014-0106-2-S3.pdf]

Additional File 3

Model of Hampered Movement onto Structures and Slowing Down

To substantiate the assertions that

- slowing down particles in a certain spatial region leads to accumulation of particles there, i.e. a higher concentration than outside these regions,
- hampering movement of particles into a certain region (i.e., letting them enter with a certain probability < 1) leads to lower concentrations of particles in these regions,
- and these two mechanisms simultaneously can lead to a homogeneous distribution of particles, i.e. no difference in concentration inside and outside the regions,

we simulated a model with two species, particles and (immovable) regions representing surface structures, and the equivalent of the following two rules.

```
Particle() + Structure() -> Structure()[Particle(diffusion:pDiff*slowdown)] @ pMoveOntoStruct
Structure()[Particle()] -> Particle(diffusion:pDiff) + Structure() @ 1
```

Simulation Settings

We used nine square regions of size 2×2 (arbitrary units) placed in a square grid with distance 2 in a system of total size 11×11, as was used for the micro-pillared surface structure simulations in the paper. The key parameters $pMoveOntoStruct$ and $slowdown$ were varied from 0.1 to 1.0 in steps of 0.1. Other parameters were also varied: the particle size was 0.01, 0.0031 or 0.001, while the number of particles in the system (initially outside the structures) was 500, 1000 or 2000. For each parameter combination, three simulations with different initial placement of particles (i.e. replications) were run for long enough to reach a steady state with respect to the concentration of particles on structures.

Results

Only the two key parameters were found to be correlated with the fraction of particles on structures at the end of the simulations runs. Hence, in the following we consider average values of all runs for each key parameter combination (i.e. we lump runs with different particle size and amounts together). It became then indeed obvious that when $pMoveOntoStruct$ and $slowdown$ were the same, the distribution of particles was roughly homogeneous.

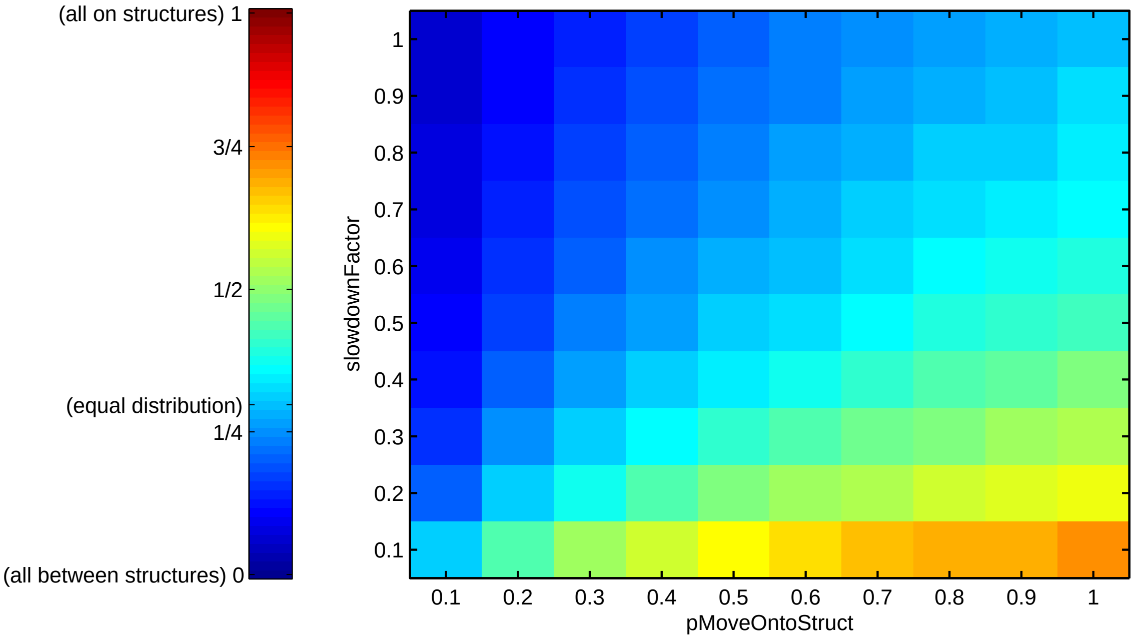

Figure A1: **Fraction of particles on structures relative to total particle number when varying the two key parameters in steps of 0.1.**

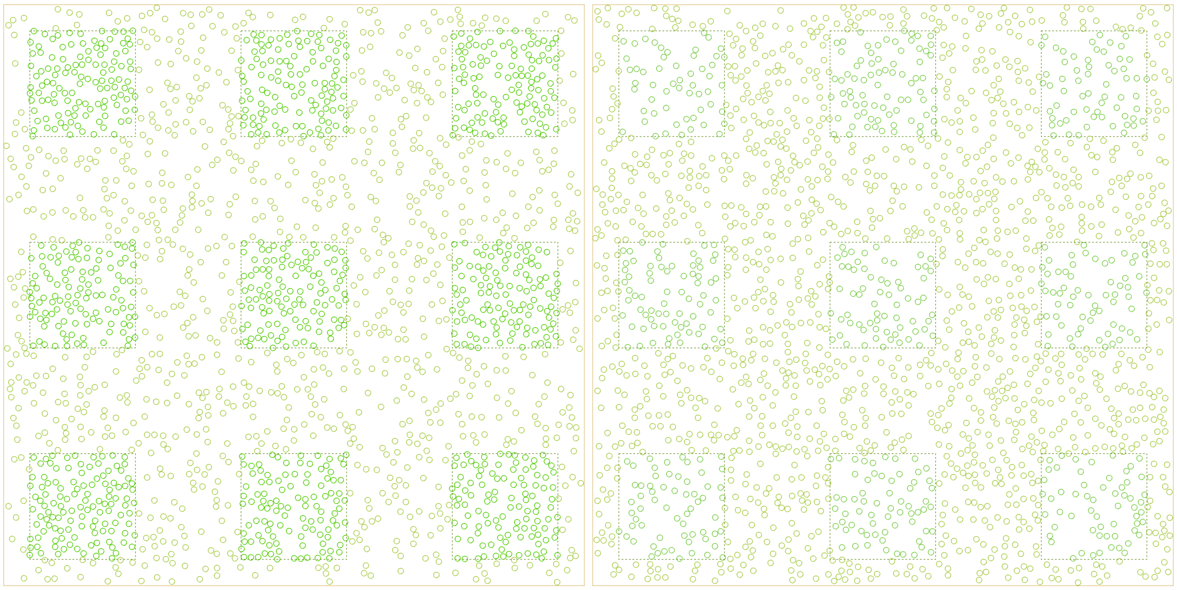

Figure A2: **Resulting particle distributions for example simulations with different settings.** Left: particle accumulation on structures with strong slowdown only and "free" entry, i.e. $pIntOntoStruct = 1$ and $intSlowdown = 0.1$, right: homogeneous distribution when using $pIntOntoStruct = 0.4$ and $intSlowdown = 0.5$.
